# An international policy on returning genomic research results

**DOI:** 10.1186/s13073-021-00928-5

**Published:** 2021-07-15

**Authors:** Anna C. F. Lewis, Bartha Maria Knoppers, Robert C. Green

**Affiliations:** 1 E J Safra Center for Ethics, Harvard University, 124 Mount Auburn Street, Suite 520 N, Cambridge, MA 02138 USA; 2 Department of Medicine, Brigham and Women’s Hospital, Boston, MA USA; 3 Harvard Medical School, Boston, MA USA; 4 Centre of Genomics and Policy, McGill University, Montreal, Canada; 5 Ariadne Labs, Boston, MA USA; 6 Broad Institute, Cambridge, MA USA

## Abstract

The Global Alliance for Genomics and Health has approved a policy for the return of clinically actionable genomic research results, the first such policy approved by an international body. The policy acknowledges the potential medical benefits to millions of individuals who are participating in genomics research. It ties the pace of implementation to each country’s clinical standards, including for the return of secondary findings, and urges funders to set aside resources to support responsible return.

Many countries and international bodies have laws and regulations that require the return of at least some genomic research results under some circumstances to participants who wish to receive them [1]. This return is supported by numerous ethical arguments, ranging from straightforward appeals to beneficence to rights-based approaches [2]. These positions focus on results that indicate risk for or the presence of a medical condition for which prevention or treatment is available (“clinically actionable” results), and for this reason, the Global Alliance for Genomics and Health (GA4GH) policy is focused on these [3]. There are countervailing opinions about the advisability of returning genomic results to research participants. These include concerns about risk-to-benefit and cost-to-benefit tradeoffs [4], as well as the argument that a strong duty to return results conflates the priorities of research with those of the clinical domain [5]. There are multiple nuances to this debate [6].

The primary mission of GA4GH, enabling data sharing, will be facilitated by an internationally relevant return of research results policy. When data is shared it will become increasingly important to understand whether and how the researchers who gathered the data responsibly considered the return of results to participants. This is particularly pertinent when data are shared across jurisdictional boundaries. In September 2020, a workshop organized by the GA4GH Regulatory and Ethics Workstream (REWS) attracted over 80 participants and reinforced the need for such a policy. A working group was convened to draft a policy with representation from multiple countries, from diverse academic disciplines, and from industry. Following working group input and REWS approval, a version of the policy was posted for a two-week public comment period on February 15. Hundreds of comments and suggestions were received online. Additionally, a discussion was held at GA4GH Connect, an open meeting. A version of the policy incorporating further open public comment was approved by the REWS in May and the Steering Committee on June 24, 2021 [3]. The policy document contains a Context section and Discussion, in addition to the following policy points:

1. *A clear protocol that is adhered to.* Every research study generating clinically actionable genomic research results should have a specific protocol regarding the return of such results. Such a protocol should be devised before the start of the study, should be approved by the relevant research oversight body, and be revisited periodically throughout the duration of the study. Researchers should be held accountable by their participants, funders, and ethics committees to meet the standards they define in this protocol.
2. *Upfront resourcing.* Where return of clinically actionable genomic results is intended, resourcing should include funding for the full process of returning results to participants, as well as ensuring the availability of appropriately trained personnel.
3. *Link to clinical standards.* In deciding the parameters for return of clinically actionable genomic results, researchers should be guided by current practice regarding the clinical standard of care within their jurisdictions.
4. *Community engagement.* Which genomic research results should be returned and how they are returned will be project and community specific, and depending on the nature of the research should be guided by community involvement.
5. *Sharing of resources.* Whenever feasible, medical, behavioral, and economic tools and outcomes associated with the return of clinically actionable genomic research results should be documented and shared to continue to lower the barriers to responsible return.
6. *Funders urged to support the return of results.* Funders should set aside resources to support the return of results in those projects that plan to do so.

As the co-chairs of the working group, we view two of these six policy points as key to the achievement of consensus and expand upon these here.

Policy point 3 states that researchers should be guided by local clinical guidelines. We acknowledge that research and clinical care represent different domains and that linking the standards in clinical care to the conduct of research represents a conflation of the two. But we judge it appropriate for three reasons. First, and most importantly, it is the prospect of a *clinical* action that motivates the return of the results in the first place. Second, existing clinical guidelines reflect many of the broad array of contextual features—such as the existence of downstream care, payment structures, and regulatory landscape—that make different policies appropriate for different locales. And finally, there is a continuum of settings encompassing clinical care, clinical trials, research studies embedded in health systems, other research studies, and population biobanks. Harmonization across these settings could lead to greater consistency and coherence.

In the clinical setting of diagnostic exome/genome sequencing, the American College of Medical Genetics and Genomics recommends the search for a defined set of clinically actionable findings that have recently been further reified in the issuance of version 3 [7, 8]. The European Society of Human Genetics has maintained that such opportunistic screening should not be performed, because of the unclear benefit-to-risk and cost-to-benefit tradeoffs [9]. In the many discussions of the GA4GH policy development, which involved REWS members as well as other individuals who responded to the public call for engagement, opinions about what was appropriate in the research setting tended to follow a similar divide, with US-based individuals favoring a more robust return of results approach, while European, Canadian, and Australian individuals emphasizing the unclear cost-to-benefit tradeoffs. Given the close relevance of clinical standards to the return of research results and the very diverse clinical contexts worldwide, these differences necessarily inform the arguments around the return of genomic results policy in the research arena. Linking the evolution of genomics return of results in research to clinical guidelines, which includes secondary findings in indication-based genomic testing, helped achieve consensus.

Policy point 6 encourages funders to support projects that wish to return results. Adoption of this policy point helped achieve consensus because while the policy does not suggest that any research project should yet be compelled to return results, it does indicate strong support for their return. Previous GA4GH work emphasized the need to explicitly include funding to enable data sharing in research budgets, and urged funders to support this as a central component of enabling all to benefit from genomics. Similarly, data generated from studies that return results to participants is key to bringing the fruits of genomic research into the clinic so that all can benefit.

Millions of individuals have already undergone some form of genomic testing in research projects. Somewhere between 1 and 3% of these carry variants that are associated with devastating but preventable health conditions [10]. As genomics research becomes even more ubiquitous, we hope that this policy, the first statement on the return of genomic research results issued by an international body, can support researchers in defining and evolving their strategies for the return of potentially life-saving information to research participants who have generously donated their DNA to science.
